# Using the Electronic Nose to Identify Airway Infection during COPD Exacerbations

**DOI:** 10.1371/journal.pone.0135199

**Published:** 2015-09-09

**Authors:** Hanaa Shafiek, Federico Fiorentino, Jose Luis Merino, Carla López, Antonio Oliver, Jaume Segura, Ivan de Paul, Oriol Sibila, Alvar Agustí, Borja G Cosío

**Affiliations:** 1 Department of Respiratory Medicine, Hospital Universitario Son Espases. IdISPa. Palma de Mallorca, Spain; 2 Department of Chest Diseases, Faculty of Medicine, Alexandria University, Alexandria, Egypt; 3 Electronic Systems Group, University of the Balearic Islands (GSE-UIB), Palma de Mallorca, Spain; 4 Department of Microbiology, Hospital Universitario Son Espases. IdISPa. Palma de Mallorca, Spain; 5 Department of Respiratory Medicine, Hospital de la Santa Creu i Sant Pau, Institut d’Investigació Biomédica Sant Pau (IIB Sant Pau), Barcelona, Spain; 6 Thorax Institute, Hospital Clinic, IDIBAPS, University of Barcelona, Barcelona, Spain; 7 CIBER de Enfermedades Respiratorias (CIBERES), Madrid, Spain; University of Athens Medical School, GREECE

## Abstract

**Background:**

The electronic nose (e-nose) detects volatile organic compounds (VOCs) in exhaled air. We hypothesized that the exhaled VOCs print is different in stable vs. exacerbated patients with chronic obstructive pulmonary disease (COPD), particularly if the latter is associated with airway bacterial infection, and that the e-nose can distinguish them.

**Methods:**

Smell-prints of the bacteria most commonly involved in exacerbations of COPD (ECOPD) were identified *in vitro*. Subsequently, we tested our hypothesis in 93 patients with ECOPD, 19 of them with pneumonia, 50 with stable COPD and 30 healthy controls in a cross-sectional case-controlled study. Secondly, ECOPD patients were re-studied after 2 months if clinically stable. Exhaled air was collected within a Tedlar bag and processed by a Cynarose 320 e-nose. Breath-prints were analyzed by Linear Discriminant Analysis (LDA) with “One Out” technique and Sensor logic Relations (SLR). Sputum samples were collected for culture.

**Results:**

ECOPD with evidence of infection were significantly distinguishable from non-infected ECOPD (*p* = 0.018), with better accuracy when ECOPD was associated to pneumonia. The same patients with ECOPD were significantly distinguishable from stable COPD during follow-up (*p* = 0.018), unless the patient was colonized. Additionally, breath-prints from COPD patients were significantly distinguished from healthy controls. Various bacteria species were identified in culture but the e-nose was unable to identify accurately the bacteria smell-print in infected patients.

**Conclusion:**

E-nose can identify ECOPD, especially if associated with airway bacterial infection or pneumonia.

## Introduction

Exacerbations of Chronic Obstructive Pulmonary Disease (ECOPD) are important events in the natural history of the disease because they influence disease progression, morbidity [1] and mortality [2]. The diagnosis of ECOPD relies on the increase of respiratory symptoms reported by the patient with or without an increase in sputum volume or purulence [3, 4]. Hence, despite the high sanitary burden of ECOPD, their diagnosis and treatment are currently empirical [3, 5].

ECOPD are characterized by a burst of pulmonary and systemic inflammation [6], generally believed to be the result of viral and/or bacterial airway infections [7, 8]. However, potential pathogenic micro-organisms (PPM) can be recovered during ECOPD in only a proportion of patients (30% of sputum cultures and 50% of bronchial secretion cultures) [9, 10]. These percentages increase slightly (59%) if quantitative PCR is used [11]. Besides, PPB can also be recovered in a proportion of clinically stable COPD patients [8]. Therefore, novel methods capable of identifying more precisely the role of infection during ECOPD are needed. They can contribute to improve the quality of care provided to these patients since they may foster a more appropriate use of antibiotic therapy under these circumstances.

Over the past decade there has been increasing interest on the potential diagnostic value of volatile organic compounds (VOCs) exhaled in human breath [12]. The electronic nose (e-nose) constitutes an emerging non-invasive technique capable of detecting and differentiating VOCs patterns (“smell prints”) in humans (“breatheomics”) [13, 14]. Recent studies have shown that the e-nose can reliably identify patients with bronchial asthma [15], lung cancer [16], bacterial pneumonia [17] and bacterial sinusitis [18], as well as to identify and classify various bacterial species cultured *in vitro* [19, 20]. In a pilot study, our group previously showed that the e-nose can detect bacterial colonization in clinically stable patients with COPD [16, 21].

Considering all these previous observations we hypothesized that the VOCs smell prints will be different during ECOPD *vs*. clinical stability, will discriminate infectious *vs*. non-infectious ECOPD or pneumonia, and will identify the specific bacterial species present in the airways during ECOPD. Accordingly, in this study we sought to: *(1)* determine the sensitivity, specificity, positive predictive value and negative predictive value of the e-nose in the bacteriological diagnosis of ECOPD with or without pneumonia which is our primary objective; *(2)* investigate whether the VOCs smell print changes in the transition from ECOPD to clinical stability; *(3)* explore the feasibility of developing a system capable of identifying and establish the presence of bacterial infection in an exhaled air sample by analysing libraries of bacteria previously identified and characterized *in vitro*.

## Methods

### Study design and Ethics

**Fig 1:**
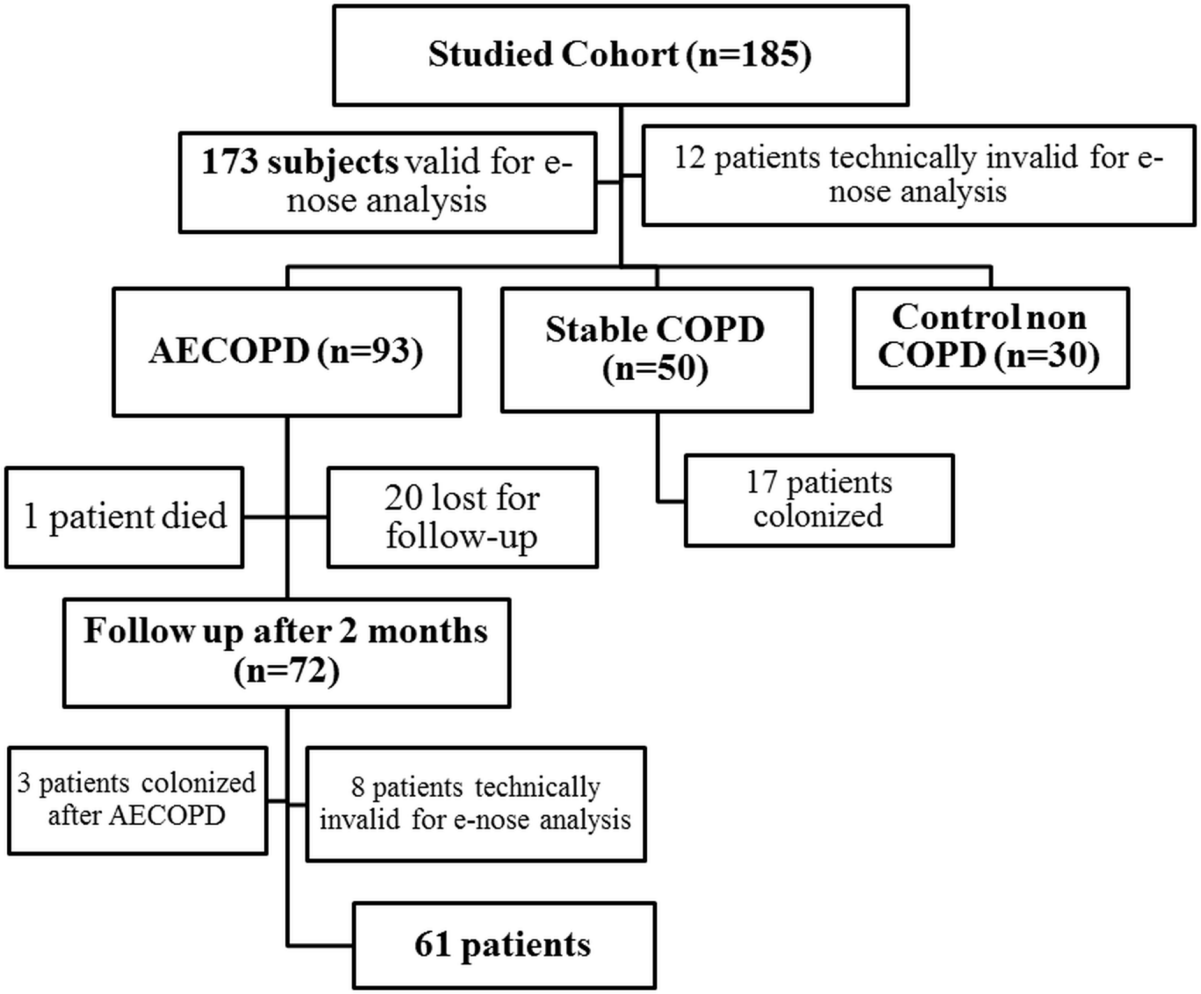
Flow chart of the studied population at every visit.

The current study was divided into 2 parts. The first part is a cross-sectional descriptive, case-controlled study that included COPD patients hospitalized because of ECOPD (with and without pneumonia) and clinically stable COPD patients as well as healthy control subjects. The second part is a longitudinal study that compared ECOPD patients (1^st^ visit) and the same ones at clinical stability two months later (2^nd^ visit); Fig 1. The sample size was calculated using a two sided Fisher exact’s test for independent case-control study with a significance level (α) of 0.05 and power of test of 80% considering that PPM growth in sputum could be detected in 30% of the patients with ECOPD [22, 23] and based on our pilot study published by Sibila et al [21]. All the patients were recruited from the ward (ECOPD), out-patient clinic (stable COPD) and lung function laboratory (controls) at Son Espases University Hospital in Palma de Mallorca (Spain). All of the participants signed their informed consent, and the study protocol was approved by the local Ethics Committee of Balearic Islands.

### Study population

COPD was defined by a post-bronchodilator FEV_1_/FVC<70% and history of tobacco consumption >10 pack-year following GOLD recommendations [5]. We included in the study patients with a diagnosis of ECOPD according to GOLD guidelines [5] defined by acute worsening of daily respiratory symptoms-beyond the normal day to day variations- that required a change in medication; ECOPD patients that had associated pneumonia proved by the presence of new infiltrates on chest radiography [24] were also included. These patients were compared with clinically stable COPD defined by absence of an exacerbation requiring antibiotic or steroid therapy within 30 days prior to inclusion and control healthy subjects with normal lung function recruited from our pulmonary function unit (Fig 1). Infective episode of ECOPD was defined by the presence of PPM growth in sputum culture (see below) in patients fulfilling the previous definition of ECOPD. Colonization was defined by the presence of PPM growth in stable COPD patients. Patients with no PPM growth in sputum culture were considered non-infected for the study purpose. All ECOPD were followed up and studied again 2 months after exacerbation, when clinically stable.

Patients with a history of asthma, primary bronchiectasis, lung cancer, active tuberculosis, interstitial lung diseases, active infection other than those of pulmonary origin including upper airways infection, and those who had taken antibiotics in the last seven days were excluded from the study.

#### Characterization of participants

We obtained in all patients a detailed clinical history (including age, gender, smoking history, relevant comorbid conditions, number of previous exacerbations, and drug history), a chest X-ray, pulmonary function measurements (including forced spirometry, carbon monoxide diffusing capacity (DLCO) and static lung volumes) following international recommendations [25] and using reference values of a Mediterranean population [26]. Chest Computed Tomography (CT) was performed if indicated clinically. Spontaneous sputum and exhaled air samples were collected consecutively in all patients.

### E-nose measurements

Exhaled breath gas was collected for e-nose assessment as previously described [15, 27]. Briefly, the exhaled gas from all subjects −as well as clear air collected from the same environment as baseline reference− were collected in a 5 Liters Tedlar bag after 3 minutes of tidal breathing through a Hans-Rudolph valve with an expiratory silica reservoir exposed to dry air and an inspiratory filter. The e-nose device (Cyranose 320; Smith Detections, Pasadena, CA), with 32 organic polymeric nano-composite sensor arrays, was then connected to the Tedlar bag as five to six measurements of the each sample were obtained over 5 minutes duration. Changes in the nanosensor’s electrical resistance between clear air and subjects’ exhaled breath generate a breath-print VOCs profile. As a difference from previous publications, the resistance value was monitored during the whole measuring time using several parameters (as slope values) to generate the breath-print profile. Transcutaneous oxygen saturation (SaO_2_) was continuously monitored during the procedure.

### Sputum culture

Spontaneous sputum samples were collected within 24 hours of presentation of ECOPD symptoms as well as from clinically stable COPD cases. All the sputum samples were collected in sterile containers and transferred to the microbiological laboratory within 2 hours of collection for Gram staining and bacterial culture. All the sputum samples were classified according to Murray-Washington criteria, thus grade IV (10–25 epithelial cells and > 25 leucocytes per field) and grade V (< 10 epithelial cells and > 25 leucocytes per field) were cultured quantitatively and a bacterial load ≥10^6^ colony forming units (CFU)/ml was considered as significant. Culture was performed in non-selective (sheep blood agar, chocolate agar) and selective media (MacConkey agar) at 37°C for 48 hours in 5% CO_2_, and bacterial species identification was conducted biochemically (Microscan, Siemens). The identified microorganisms were classified as potentially pathogenic microorganisms (PPM) including *Streptococcus pneumoniae*, *Haemophilus influenzae*, *Pseudomonas aeruginosa*, *Staphylococcus aureus* methicillin susceptible or resistant, *Moraxella catarrhalis*, and Gram negative-bacilli, and non-PPM included *Candida spp*., *Streptococcus viridans*, *Neisseria spp*., *Staphylococcus epidermidis* and *Corynebacterium spp*.

### In vitro e-nose learning

**Fig 2:**
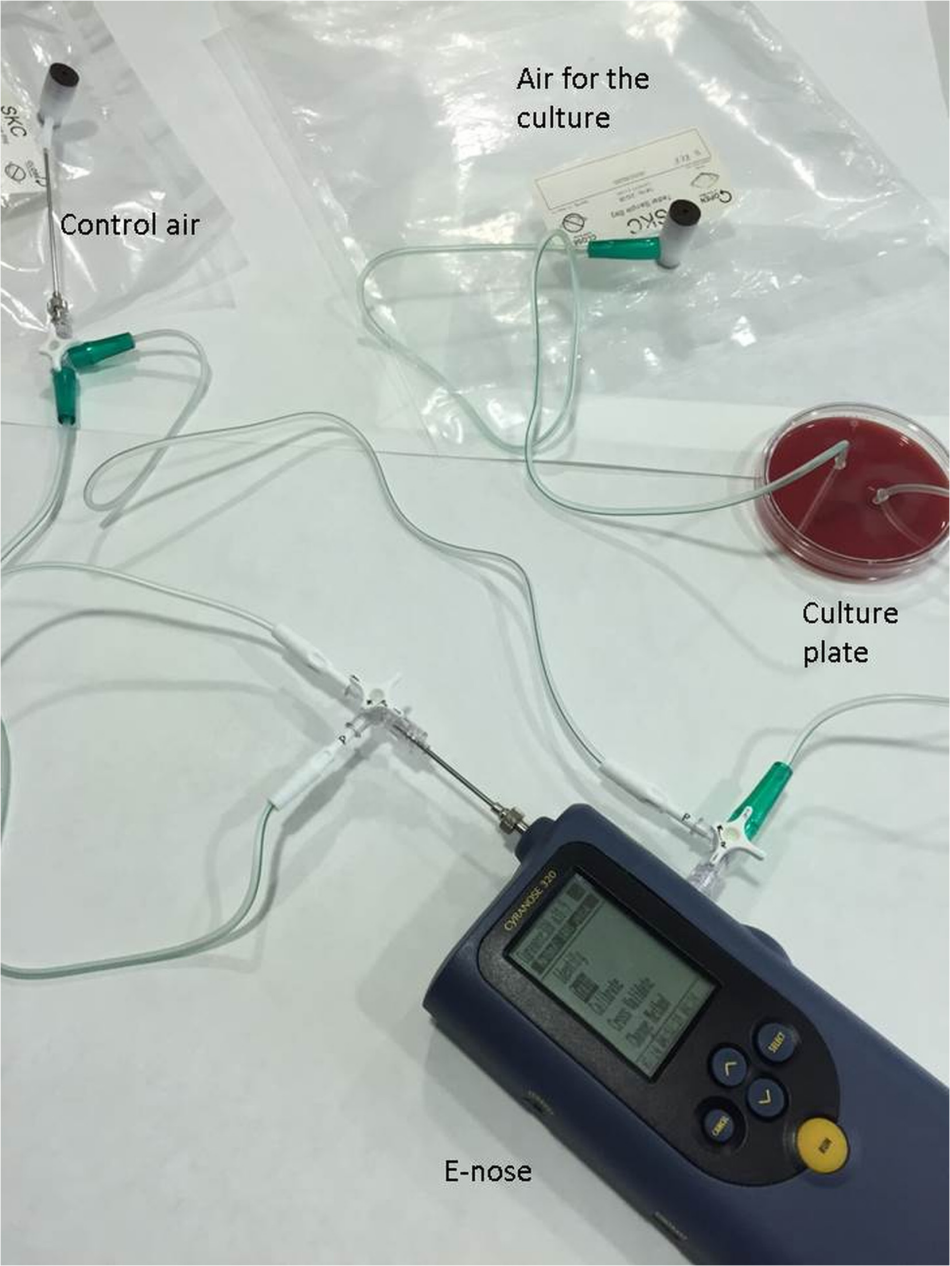
Identification of bacteria *in vitro*. Experimental setting.

To let the e-nose learn the breath print of specific bacteria, at least one representative clinical isolate for each species included in the potentially pathogenic microorganism group was analyzed by using the e-nose, specifically 3 *S*. *pneumoniae*, 3 *H*. *influenzae*, 3 *P*. *aeruginosa*, *1 S*. *aureus*, 1 *M*. *catarrhalis*, and 1 *E*.*coli* isolate were included (Table A in S1 File). These bacteria were cultured on appropriate non-selective media (sheep blood agar and/or chocolate agar) at 37°C for 18–24 hours in 5% CO_2_ on Petri plates. Then, plates were exposed to e-nose inhalation to build up a library of VOCs pattern for each bacteria following previously described methodology [19]. Briefly, filtered air from a Tedlar bag was introduced into the close plates through a hole located in one extreme of the plate cover; then the VOC were extracted and delivered to the e-nose through a second hole located in the other side of the plate cover (Fig 2). Several exposition times were tried and finally an exposition time of about 6 minutes was selected as it coincides with no changes in the slope of the sensors. The e-nose sensors registered continuously the relative changes in resistance due to volatile organic compounds in the headspace (i.e., the space over the agar media in the Petri plates) using mathematical algorithms for each bacterium independently on the culture media used. Each measure was compared with a reference measure to obtain the sensor readout, being the reference a non-inoculated Petri plate. For each plate of the different bacterial strains, the measurement was repeated eight times with a total duration of 20 minutes.

### Data analysis

Results are presented as mean ± standard deviation (SD) for quantitative variables or absolute numbers and relative frequencies for categorical variables. Comparisons between different groups were analysed with the Chi Square, Mann-Whitney or Kruskal-Wallis tests as appropriate. MedCalc (version 9.2.1.0, Acacialaan 22, B-8400 Ostend, Belgium) was used for analysis.

Breath-print data from ECOPD patients (infected and non-infected), ECOPD with associated pneumonia, stable COPD as well as healthy controls were analysed using two methods: Linear Discriminant Analysis (LDA) for comparison between the different groups [15] and Sensor Logic Relations (SLR), a new data management approach to identify different microorganisms in exhaled breath. Briefly, instead of considering the values of the relative resistance to generate the breath-print for each patient, breath-print is built with Logic Relations between the Resistor values as “Resistor 2 value is greater than Resistor 5”. This technique is adopted to consider the relative sensitivity of two sensors to a certain VOC profile (additional information provided in S1 File). The results were expressed as success classification ratio [28] as well as sensitivity and specificity.

## Results

### Population characteristics

We included in the study 185 subjects, 93 of whom had a diagnosis of ECOPD (19 of them with associated pneumonia), fifty patients with clinically stable COPD and 30 control healthy subjects (Fig 1).

Table 1 presents the main demographic and clinical characteristics of the population studied. COPD patients-either ECOPD or clinically stable- were mostly males, with a similar age and proportion of co-morbidities. Cumulative smoking exposure (pack-years) was higher in COPD patients than healthy controls; however the ex-smokers constituted the highest proportion among all groups (Table 1). COPD patients had moderate to severe airflow limitation and reduced DLCO whereas controls had normal lung function (Table 1). Previous admissions were higher in the ECOPD group compared to stable COPD patients and healthy controls. Chest CT scanners were available for 108 participants. Emphysematous changes were detected in > 50% of all COPD patients and in 13.3% of smoker controls. Bronchiectasis was detected only among COPD groups being more prevalent in ECOPD either with or without associated pneumonia (20.5% and 15.7% respectively; Table 1) than stable COPD patients.

**Table 1:** Demographic, clinical and functional characteristics of the population studied.

Variable		ECOPD(n = 74)	ECOPD with pneumonia(n = 19)	Stable COPD(n = 50)	*p* value^$^	Control non-COPD (n = 30)	*p* value^#^
**Age** (years)		68 ± 7.8	70 ± 9.3	68.6 ± 9.5	0.699	63.8± 12.4	0.291
**Gender** (M/F); n		53/21	16/3	38/12	0.534	13/17	0.033*
**Smoking**; n (%)							
	Current smoker	33 (44.6)	11 (57.9)	24 (48)		4 (13.3)	
	Ex-smoker	41 (55.4)	8 (42.1)	26 (52)	0.659	14 (46.7)	<0.0001*
	Non-smoker					12 (40)	<0.0001*
	Pack/years	58.4±26.1	58.6 ± 29.4	61.6 ± 37.4	0.976	15.5± 18.3	<0.0001*
**Comorbidity**; n (%)		57 (77)	12 (63.2)	42 (84)	0.427	20 (66.7)	0.743
**Previous admission**		3.9 ± 3.8	3.1 ± 3.3	1.8 ± 2.9	0.002*	0.13± 0.35	<0.0001*
**Lung Function**							
	FEV1/FVC, %	43.2 ±11.9	44.1 ±13.02	47.1 ±13.7	0.326	77.2 ± 5.5	<0.0001*
	FEV1 (L)	1.2 ± 0.5	1.4 ± 0.5	1.6 ± 0.8	0.059	2.5 ± 0.7	<0.0001*
	FEV1% ref.	47.3 ±16.7	53.9 ± 26	56.4 ± 22.6	0.166	98.5± 13.4	<0.0001*
	FVC (L)	2.9 ± 0.9	3.1 ± 0.9	3.3 ± 0.97	0.249	3.2 ± 0.8	0.369
	FVC % ref.	84.9 ± 22.6	90.7 ± 27.8	89.08 ± 18.8	0.603	98.5 ±12.5	0.007*
	DLCO% ref.	46.6 ± 17.4	42.3 ± 22.2	50.9 ± 15.3	0.252	78.7 ± 9.5	<0.0001*
	KCO% ref.	55.5 ± 24.03	40.1 ± 20.2	56.8 ± 14.3	0.074	79.4 ± 8.5	<0.0001*
**Chest CT**; n (%)							
	Emphysema	45 (60.8)	10 (52.6)	27 (54)	0.424	4 (13.3)	0.001*
	Bronchiestasis	15 (20.2)	3 (15.7)	7 (14)	0.126	0 (0)	0.013*
**sputum PPM**; n (%)		34 (46)	8 (42.1)	17 (34)	NA	0 (0)	NA
**SaO_2_ before breath collection**		93.9 ± 2.6	92.6 ± 3.3	94.9 ± 2.3	0.630	96.9 ± 0.9	0.773
**SaO_2_ after breath collection**		93.2 ± 3.3	91.7 ± 3.02	95.4 ± 2.8		96.9 ± 1.3	

Abbreviations: M/F: male/female, SAO_2:_ oxygen saturation, NA: not assessed

^$^ p value for comparing between ECOPD, ECOPD with pneumonia and stable COPD

^#^ p value for comparing the 4 groups.

### Microbiological cultures

Forty-two patients (45%) with ECOPD and seventeen (34%) of stable COPD had PPM in the baseline characterization (Table 1 and S1 Fig A in S1 File). Sputum samples did not show growth of PPM in 19% of exacerbation episodes and were considered as non-infective episodes. *Pseudomonas aeruginosa* was the most commonly isolated microorganism (34%) followed by *Haemophilus influenzae* (13.6%), *Streptococcus Pneumoniae* (10.2%), *Moraxella catarrhalis* (8.5%) then *Staphylococcus aureus* methicillin susceptible (6.8%). Other species of bacteria were isolated in 23.7% and *Candida spp*. was isolated in 10.2%.

### Breath-print analysis

Exhaled-breath air collection during the ECOPD was safe without evidence of significant desaturation (*p* = 0.773, Table 1). The breath-prints from different COPD groups, either exacerbated or stable, were statistically discriminated from those obtained from healthy control subjects (*p* < 0.05, Table 2).

**Table 2:** Percentage of success ratio, sensitivity and specificity of the e-nose when comparing different groups of patients and controls in the presence or absence of PPM in sputum.

	Absence of PPM in sputum				Presence of PPM in sputum			
	% of success ratio	*P*	Sn.	Sp.	% of success ratio	*P*	Sn.	Sp.
Stable COPD *vs* Controls	72%	**0.041**	72%	70%	72%	**0.005**	70%	%73
ECOPD *vs* Controls	74%	**0.041**	66%	80%	74%	**0.001**	68%	80%
Pneumonia *vs* Controls	87%	**0.006**	75%	90%	97%	**0.005**	88%	100%
ECOPD *vs* Pneumonia	86%	**0.021**	85%	86%	88%	**0.005**	91%	75%
ECOPD *vs* Stable COPD	76%	**0.018**	89%	48%	64%	0.074	57%	69%
Stable COPD *vs* Pneumonia	91%	**0.021**	95%	63%	86%	**<0.001**	88%	75%

Sn: sensitivity, Sp: specificity.

#### ECOPD compared to stable COPD

In the cross-sectional analysis, breath-prints from ECOPD were significantly distinguishable from stable COPD in case of absence of PPM (*p* < 0.05; Table 2). Breath-prints of stable COPD patients with evidence of PPM, indicating the presence of airway colonization, were not statistically different from infective ECOPD (*p* = 0.074; Table 2).

In the longitudinal analysis, when the same patient was compared between exacerbation and stability, the e-nose was able to distinguish breath-prints during ECOPD (1^st^ visit) from clinical stability (2^nd^ visit) with a classification success ratio of 70%, sensitivity of 72% and specificity of 67% (Table 3). Stable COPD patients during the 2^nd^ visit with evidence of PPM in sputum were excluded from the analysis.

**Table 3:** Percentage of success ratio, sensibility and specificity of the e-nose when comparing different groups of patients based on the presence (infected) or absence of PPM (not infected) in sputum and the exacerbated or stable condition.

	% of success ratio	*p* value	Sn	Sp
ECOPD (not infected-infected)	75%	**0.018** *	88%	60%
Pneumonia (not infected-infected)	100%	**0.014** *	100%	100%
ECOPD not infected–All groups infected	68%	**0.026** *	81%	59%
Infected by Pseudomonas–Other infections^**$**^	56%	0.196	55%	56%
Infected by Pseudomonas–Not infected^**$**^	68%	0.12	45%	70%
ECOPD with Pseudomonas–ECOPD not infected	69%	0.37	66%	71%
1^st^ visit ECOPD– 2^nd^ visit ECOPD	70%	0.068	74%	67%

^**$**^ Comparison done irrespective of patient group

* significant *p* value < 0.05

#### Pneumonia vs. non-pneumonia patients

**Fig 3:**
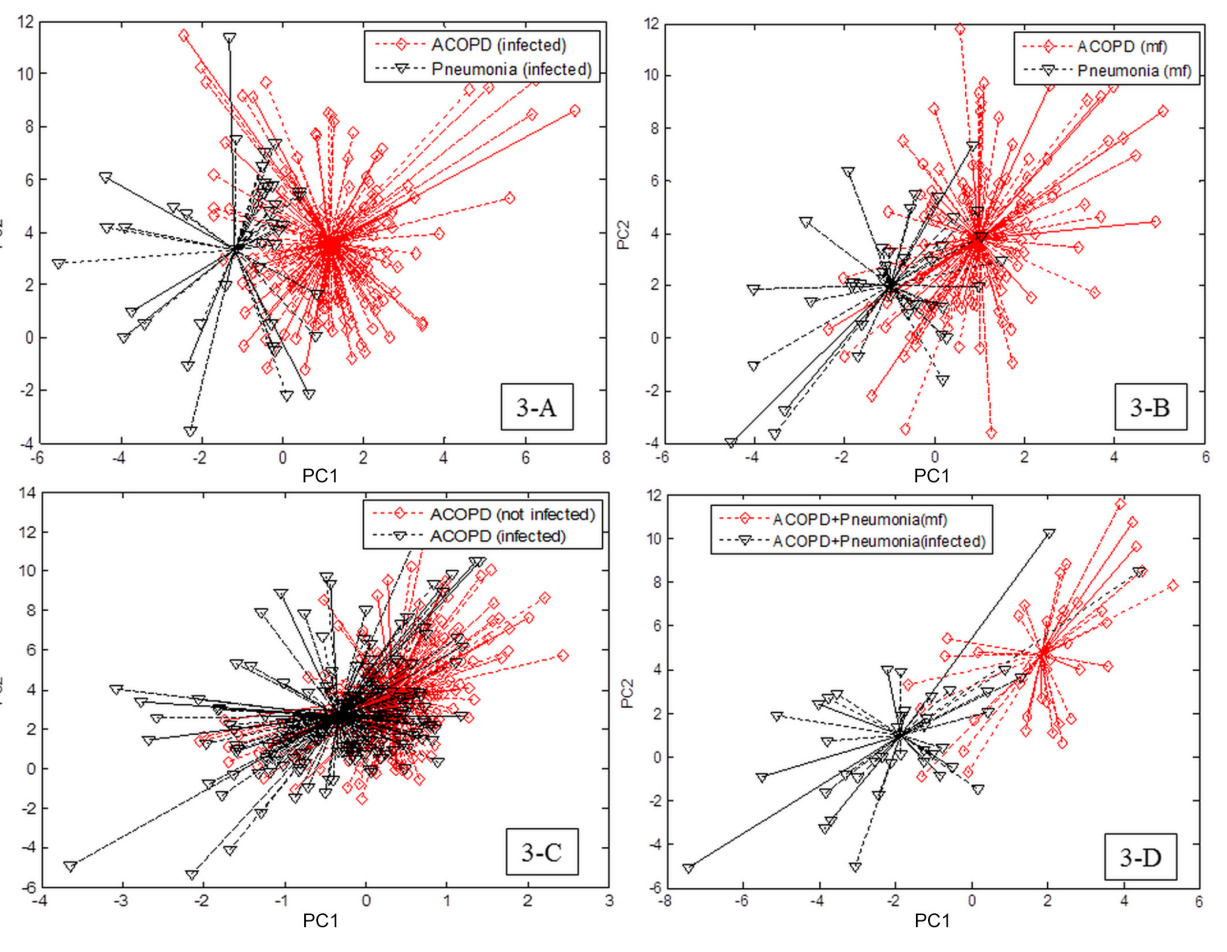
Two-dimensional principal component (PC) analysis plots: showing breath-prints discrimination between ECOPD versus ECOPD with pneumonia in case of infection (A) and absence of infection (B); ECOPD with infection versus ECOPD without infection (C); ECOPD versus ECOPD with pneumonia without infection (D).

Breath-prints from pneumonic ECOPD were significantly distinguishable from stable COPD (classification success ratio: 86%, with sensitivity of 88% and specificity of 75%, *p* <0.001; Table 2). Further, the e-nose was able to significantly discriminate the breath-prints of ECOPD associated with pneumonia from non-pneumonic ECOPD regardless the presence of PPM (classification success ratio: 88%, *p* = 0.005 with a sensitivity of 91% and specificity of 75%, Table 2, Fig 3A) or absence of PPM (classification success ratio: 86%, *p* = 0.021; sensitivity = 85%, specificity = 86%; Table 2, Fig 3B).

#### Infected vs. non-infected ECOPD

Among the ECOPD, the e-nose was able to discriminate breath-prints of infected patients versus non-infected patients with a classification success ratio of 75% (*p* = 0.018), sensitivity of 88% and specificity of 60% (Table 3, Fig 3C). Moreover, the percentage of success ratio was further improved in the pneumonic subgroup of ECOPD (classification success ratio: 100%, *p* = 0.014; sensitivity and specificity of 100%; Table 3, Fig 3D).

#### Bacterial identification in vitro

**Fig 4:**
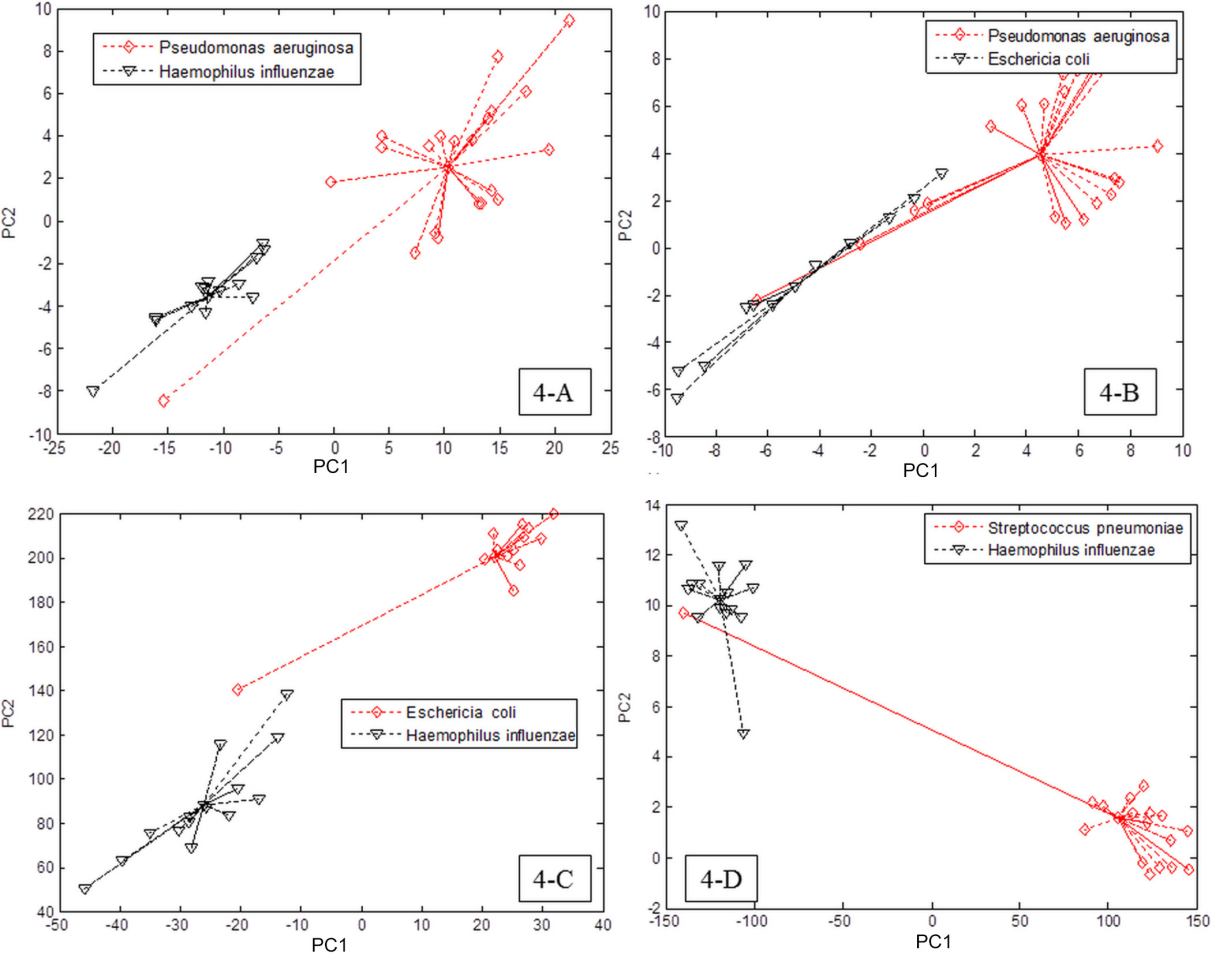
Comparison between e-nose smell-prints among different species of bacteria. The graphs shows two-dimensional principal component (PC) analysis plots showing smell-prints discrimination between 2 species of bacteria.

When comparing different species of bacteria *in vitro*, the e-nose successfully discriminated the smell-print of two single species of bacteria (*p* <0.01, Fig 4A–4D and Fig B in S1 File) using the LDA method. Upon analysing the same smell-prints using SLR, one single species of bacteria (namely *Pseudomonas aeruginosa*, *Streptococcus pneumoniae*, *Haemophilus influenzae*, and *Escherichia coli*) was successfully discriminated versus all other species with success ratios varying between 97 and 100%. Results upon single comparisons are provided in the on-line data supplement (Tables A, B, C, D and E in S1 File).

#### Bacterial identification in vivo

Despite the successful discrimination of bacteria species *in vitro*, and the effective detection of infective ECOPD, the e-nose failed to identify a breath-print of an individual bacterium in the infected patient when compared to a non-infected, except for *Haemophilus influenzae*. Where different sensitive sensors obtained during *in vitro* analysis (Tables B, C and D in S1 File) did not show similar sensitivity for analysing the same bacterial species *in vivo*, this was not the case for *Hemophilus influenza* in which sensors 14, 15 and 19 (Table 4 and Table D in S1 File) were linked to the presence of *Hemophilus influenza* both *in vitro* and *in vivo*. Moreover, two different species of bacteria could be differentiated with a success classification ratio of 82%, 95% and 100% using SLR analysis for *Pseudomonas aeruginosa* vs *Hemophilus influenzae*, *Pseudomonas aeruginosa* vs *Escherichia coli* and *Hemophilus influenza* vs. *Escherichia* coli respectively (Table 4).

**Table 4:** Success ratio and pattern of stimulated sensors when comparing two different species of bacteria in the breath samples of infected patients.

Analysis	% of success ratio	SLR utilized
PA vs HI	82	21/17; 2/19
PA vs EC	95	2/13; 12/14
CA vs HI	100	14/9; 15/12; 15/16
CA vs EC	90	21/7; 7/10
HI vs EC	100	24/7; 13/1; 22/16

Abbreviations: PA: *Pseudomonas aeruginos*, HI: *Hemophilus influenzae*, EC: *Escherichia coli*, CA: *Candida*

## Discussion

In the current study, we described for the first time the validity of the e-nose technology in the discrimination of infective episodes for ECOPD. Using simple mathematical algorithms the e-nose distinguished specific breath-prints for infected ECOPD, and was able to detect when the exacerbation was associated with pneumonia in comparison to non-infected ECOPD. Further, it was able to identify single species of bacteria *in vitro* and still could differentiate between two different groups of bacteria such as *Pseudomonas aeruginosa* and *Hemophilus influenzae in vivo*.

### Previous studies

Sputum cultures for microorganism detection are considered to be of limited value in investigating the etiology of ECOPD [9, 10]. Stolz et al [29] identified pathogenic bacteria in the sputum samples in 36% to 38% in their randomized groups of ECOPD. Recently, Boixeda et al [30] identified bacterial agent in only 24.1% of their cohort as an etiology of ECOPD, being *Pseudomonas aeruginosa* and *Haemophilus influenza* the most common detected microorganisms. Shykhon et al [31] and Lai et al [32] identified infection from the upper respiratory tract using e-nose with an accuracy of 88.2%. Thaler et al [18] reported correct e-nose diagnosis of bacterial sinusitis in 72% of their cohort. Further, Hanson et al [17] and Hockstein et al [33] succeeded to detect bacterial infection in ICU patients with ventilator associated pneumonia using e-nose with a prediction accuracy of more than 90%. Much more recently, Sibila et al [21] reported 88% accuracy of e-nose in the identification of airway bacterial colonization in stable COPD patients. Our results agree with these previous observations, as 45% of our sputum samples during ECOPD showed PPM and we were able to differentiate them from non-infective episodes with e-nose technology.

### Interpretation of results

ECOPD is generally associated with a burst of airway inflammation [6, 34, 35]. This could be the cause for a specific VOCs breath pattern in this condition, which is independent of the presence of infection. Our results confirmed this concept as we demonstrated that a specific breath pattern of ECOPD distinguishable from stable COPD by classification success ratio of 76% in the absence of PPM. Additionally, we demonstrated a significant distinct breath-print pattern of ECOPD using e-nose and clearly distinguishable from healthy controls. This also can be explained on the basis that the e-nose can clearly differentiate the COPD as a chronic inflammatory airway disease [5, 21] with special breath pattern from others. Also, Fens et al [27] and Dragonieri et al [36] discriminated COPD from asthma and from non-small cell cancer with accuracy of 96% and 85% respectively.

Interestingly, we demonstrated a specific breath pattern for infected ECOPD versus non-infective episodes with a classification success ratio of 75%. This pattern can be due to firstly the intensive airway inflammatory response and oxidative stress associated with bacterial infection in ECOPD [37, 38] that could be a direct cause of specific VOCs breath pattern easily detectable by the e-nose. Secondly, the e-nose technology is able to identify bacterial pathogens [19, 20]. This is supported by the recent results demonstrated by our previous pilot study [21] as breath-print for bacterial colonized COPD was differentiated from non-colonized with accuracy of 89%. Also, it may explain the inability of the e-nose to differentiate colonized stable patients from acutely infected during exacerbation in our analysis.

Further, the breath pattern of ECOPD was distinct from those presented with associated pneumonia either in case of positive PPM (classification success ratio of 88%) or not (classification success ratio of 86%) indicating two different acute events with higher inflammatory response in case of pneumonia [39].

According to our results, different bacterial species were clearly discriminated *in vitro* which was previously demonstrated by Dutta et al [19] and Pavlou et al [20]. Moreover, in COPD patients, the e-nose technology could differentiate between two different groups of bacteria in the exhaled breath samples using SLR; as *Pseudomonas aeruginosa* versus *Hemophilus influenzae* could be discriminated with excellent success ratio. Joensen et al [40] could differentiate cystic fibrosis patients colonized with *Pseudomonas aeruginosa* and failed to differentiate patients colonized with other pathogens. However, there was no clear link between the *in vitro* sensitive sensors used for detection of bacterial species and *in vivo* detection except partially for *Hemophilus influenza*. This could be due to high complexity of the exhaled breath which could be affected by various factors as diet, medications, endogenous metabolism, metabolism by concomitant resident and pathogen microorganisms as well as varies airway inflammatory process accompanied the ECOPD [6, 34, 35].

### Clinical implications

Our results showed that the e-nose is able to discriminate a specific exhaled breath pattern for severe ECOPD-compared to healthy control, stable COPD and COPD with pneumonia- using a noninvasive, handheld, well tolerated and rapid novel technique. Although it is not specific enough to guide antibiotic therapy, it has the potential to become a diagnostic tool for ECOPD associated to bacterial infection in daily clinical practice rather than being dependent on symptomatic diagnosis [3, 4]. Further, our study shows the ability of the e-nose to identified the infective ECOPD as well as COPD with pneumonia which help in the decision of starting antibiotics rather than being empirical or depending on the severity of exacerbation [41]. This will guard against the emergence of multidrug resistance and modulate the economic burden of antibiotic therapy on one hand and different biomarkers for diagnosing bacterial infection as a cause for ECOPD or guidance of antibiotic therapy [29, 42] on the other hand.

Further studies are needed to determine whether stratification of patients by severity or by clinical phenotype (i.e. emphysematous/non-emphysematous, frequent exacerbator, COPD/asthma overlap, etc) are able to reduce the variance of measures.

### Study limitations

Our study has some limitations; firstly, some patients were unable to produce sputum samples and they were considered as non-infective episodes, which could not be the case, and we assume that this fact could be responsible for the low specificity found. Further, we did not consider the variation of the exacerbations’ duration at assessment. Secondly, we did not search for viral aetiology as a cause of ECOPD [43] or pneumonia in our cohort. However, bacteria still constitute the commonest cause of ECOPD [7]. Thirdly, we used only Cyranose e-nose as a diagnostic tool in all our analysis. However, Lai et al [32] and Dutta et al [19] used a similar device to identify bacterial pathogens in upper respiratory infection. More recently, Joensen et al [40] and Sibila et al [21] used Cyranose to identify infection in exhaled breath in cystic fibrosis and colonized stable COPD respectively. Further, technical reproducibility and repeatability of e-nose measurements was tested previously for Cyranose e-nose by Fens et al [27] and McWilliams et al [44] with excellent correlation coefficients indicating similar sensor responses. Finally, our study detected VOC mixtures rather than identifying a specific VOC. Further, we did not measure inflammation in the sputum which is highly accentuated during ECOPD [6, 34, 35]. In order to know which compound of VOCs is associate with exacerbations and responsible to exhaled breath discrimination as well as better identification of inflammation, the application of other techniques as gas chromatography and mass spectrometry is required.

## Conclusions

The e-nose is a well-tolerated, non-invasive, and rapid technique that could be a useful tool to identify infective ECOPD episodes of bacterial aetiology. Furthermore, it is able to successfully distinguish ECOPD associated with pneumonia even if microorganism is not cultured. However, further studies are needed to improve the accuracy in detecting specific micro-organisms *in vivo* despite its consistent results *in vitro*.

## Supporting Information

S1 File Contains the following files: Table A. Database of the bacteria analyzed in the culture media; Table B. LDA results in the comparison between two different species of bacteria in the culture media; Table C. LDA results in the comparison between one species of bacteria and the other species in the culture media; Table D. SLR results in the comparison between one species of bacteria and the other species in the culture media; Table E. LDA results in the comparison between two groups of bacteria in the breath-print of the patients; Fig A. The prevalence of infection, mixed flora and invalid sputum for culture among different studied groups; Fig B. Comparison between e-nose smell-prints among different species of bacteria. The graphs shows two-dimensional principal component (PC) analysis plots showing smell-prints discrimination between 2 species of bacteria in Fig B-A and between one species and all the others in Fig B-B and C.
(DOCX)
