# Supplementary material for: Using the Electronic Nose to Identify Airway Infection during COPD Exacerbations
Source: PLoS One. 2015 Sep 9;10(9):e0135199. doi: 10.1371/journal.pone.0135199 (PMC4564204; doi:10.1371/journal.pone.0135199)
Supplement: S1 File — The graphs shows two-dimensional principal component (PC) analysis plots showing smell-prints discrimination between 2 species of bacteria in Fig B-A and between one species and all the others in Fig B-B and C. (DOCX) [file pone.0135199.s001.docx]

**SUPPORTING INFORMATION**

**Methods**

For LDA, breath-print data were analyzed using pattern-recognition application built with MATLAB software (v.R2012a) and represented by logarithmic regression either mono- or bi-dimensional graphics following published algorithms.[^1^](#_ENREF_1) Briefly, raw data were reduced by principal component analysis (PCA) to three principal factors which entered a univariate ANOVA testing followed by post-hoc least significant difference test. The patients were classified based on these PCA factors. The first classification was done using only non-infected patients. Then, the bacteria-infected patients were included in the set, eliminating randomly the same number of included non-infected patients, to have exactly the same statistical environment. Breath-prints were analyzed by LDA with “One Out” technique [^2^](#_ENREF_2) and the result shown is the classification success ratio and its sensitivity and specificity. A *p* value <0.05 for the trained discriminant function was considered statistically significant.

Regarding SLR, we analyzed binary information taken from indirect sensor measurements instead of considering the sensors resistance value as input information for pattern recognition algorithms. SLR method consists of n-bit vector generated from experimental data using two metrics defined as Sensor Relationship (SR) and Resistance Range (RR).

The SR is a relationship based on the result of two-sensor comparisons and defined as a logical value. The simplest relationship between two sensors S1 and S2 compares their absolute value and is stated as S1 > S2. In this way the SR between sensors S1 and S2 SR_1-2_ is 1 if S1 > S2, and 0 otherwise. Other logical relationships can be defined by scaling one sensor measurement like S1 > *a*·S2, with *a* being a given constant. For S sensors and N_A_ scaling factors, the total number of sensor relationships is given by N = [S·(S+1)/2]·N_A_. In our work we evaluate all the possible sensor relationships for each bacteria, and consider as valid data only the SRs providing different results for at least 10% of the measurements. SRs providing the same results for all the microorganisms in more than 90% of the cases are not useful for bacteria discrimination and will not be included in the analysis. For the RR; each sensor resistance has a given *Operation Range* (OR) for all measurements considered. Each OR is divided into 10 uniform steps, and two limits are selected randomly. The samples within these limits (within 30% - 90% of the OR) are classified as true samples, while the remaining ones are classified as false. Similarly to SR, a given RR is not considered in the analysis if it provides more than a 90% of same results for all the microorganisms. That analysis was described in a manner binary value rather than *p* value.

**Results**

**Table A: Database of the bacteria analyzed in the culture media**

| ID | **Bacteria** | **Stains analyzed** |
| --- | --- | --- |
| PA | Pseudomonas aueruginosa | 5 |
| SA | Staphylococcus aureus | 1 |
| SP | Streptococcus pneumoniae | 4 |
| CA | candida | 2 |
| EC | Escherichia coli | 3 |
| HI | Haemophilus influenzae | 3 |
| BC | Branhamella catarrahis | 1 |

**Table B: LDA results in the comparison between two different species of bacteria in the culture media**

| **Analysis** | **% of success ratio** | ***p* value** | **Sensors used** | **Other sensors** |
| --- | --- | --- | --- | --- |
| PA vs SP | 100 | 0∙07 | 11,22,23 | 7,8,9,17,18,20,21 |
| SP vs EC | 100 | **2e-9*** | 15,**16** | 2,4,6,7,9,17 |
| SP vs HI | 100 | **1e-5*** | 5,15,**18**,19,22 | 1,2,10 |
| PA vs HI | 100 | 0∙16 | 15,**18**,22 | 3,7,8,17,21,23 |
| PA vs EC | 100 | 0∙09 | **12,18,22** | 10,13,17,19 |
| EC vs HI | 100 | **7e-7*** | 1,8,19 | 3,7,10,11,20,24 |
| SP vs CA | 100 | 0∙3 | **6,21** | 7,11,14,23 |
| PA vs CA | 100 | **0∙02*** | 3,6,9,10,14,23 | 5,11,17 |
| EC vs CA | 100 | **4e-4*** | 6,**8**,13 | 3,4,10,11,12,14,16,19 |
| HI vs CA | 100 | **8e-3*** | **8** | 6,9,10,11,12,13,16,17,20,23,24 |

* Significant *p* value < 0∙01

**Table C: LDA results in the comparison between one species of bacteria and the other species in the culture media**

| **Analysis** | **% of success ratio** | ***p* value** | **Sensors used** |
| --- | --- | --- | --- |
| SP vs ALL | Cannot be discriminated | | |
| PA vs ALL | 95 | 0∙35 | 11,15,20,22 |
| CA vs ALL | Cannot be discriminated | | |
| EC vs ALL | 95 | 0∙05 | 5,12,19 |
| HI vs ALL | 100 | 0∙08 | 8,11,12,17,**18** |

**Table D: SLR results in the comparison between one species of bacteria and the other species in the culture media**

| **Analysis** | **% of success ratio** | **SLR utilized** |
| --- | --- | --- |
| SP vs ALL | 100 | 6/2; 6/22; 8/2; 11/3 |
| PA vs ALL | 97 |  |
| CA vs ALL | 100 | 4/3; 11/16; 8/10; 11/23; 15/20 |
| EC vs ALL | 100 | 4/5 |
| HI vs ALL | 100 | 14/18; 15/18; 14/19 |

**Table E: LDA results in the comparison between two groups of bacteria in the breath-print of the patients**

| **Analysis** | **% of success ratio** | ***p* value** | **Sensors used** | **Other sensors** |
| --- | --- | --- | --- | --- |
| PA vs CA | 85 | 0∙37 | 11 ; 14; 19; 21 |  |
| PA vs HI | 78 | 0∙35 | 7; 17; 21 |  |
| PA vs EC | 78 | 0∙28 | 6; 7 | 23 |
| CA vs HI | 86 | 0∙28 | 19 | 4; 9; 18 |
| CA vs EC | 90 | 0∙17 | 12; 14; 18; 20 |  |
| HI vs EC | 90 | 0∙26 | 11; 22 |  |

**References**

1. Fens N, Zwinderman AH, van der Schee MP, de Nijs SB, Dijkers E, Roldaan AC, et al. Exhaled breath profiling enables discrimination of chronic obstructive pulmonary disease and asthma. Am J Respir Crit Care Med. 2009; 180(11): 1076-82.

2. Dragonieri S, Schot R, Mertens BJ, Le Cessie S, Gauw SA, Spanevello A, et al. An electronic nose in the discrimination of patients with asthma and controls. J Allergy Clin Immunol. 2007; 120(4): 856-62.

**Figures**

**Fig.** A. The prevalence of infection, mixed flora and invalid sputum for culture among different studied groups.

**Fig.** B. Comparison between e-nose smell-prints among different species of bacteria. The graphs shows two-dimensional principal component (PC) analysis plots showing smell-prints discrimination between 2 species of bacteria in S1-A and between one species and all the others in S1-B and C.


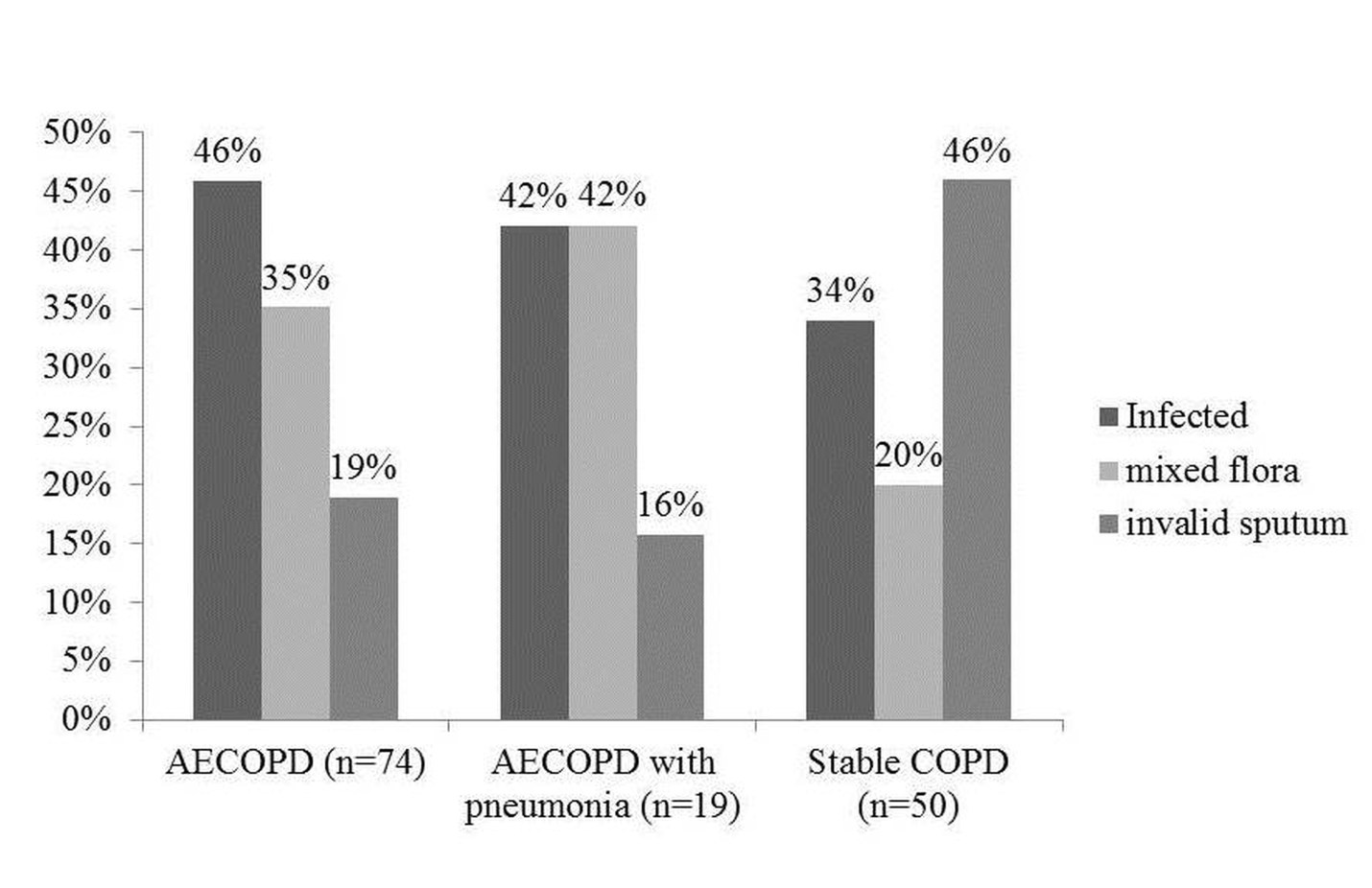


Fig. A


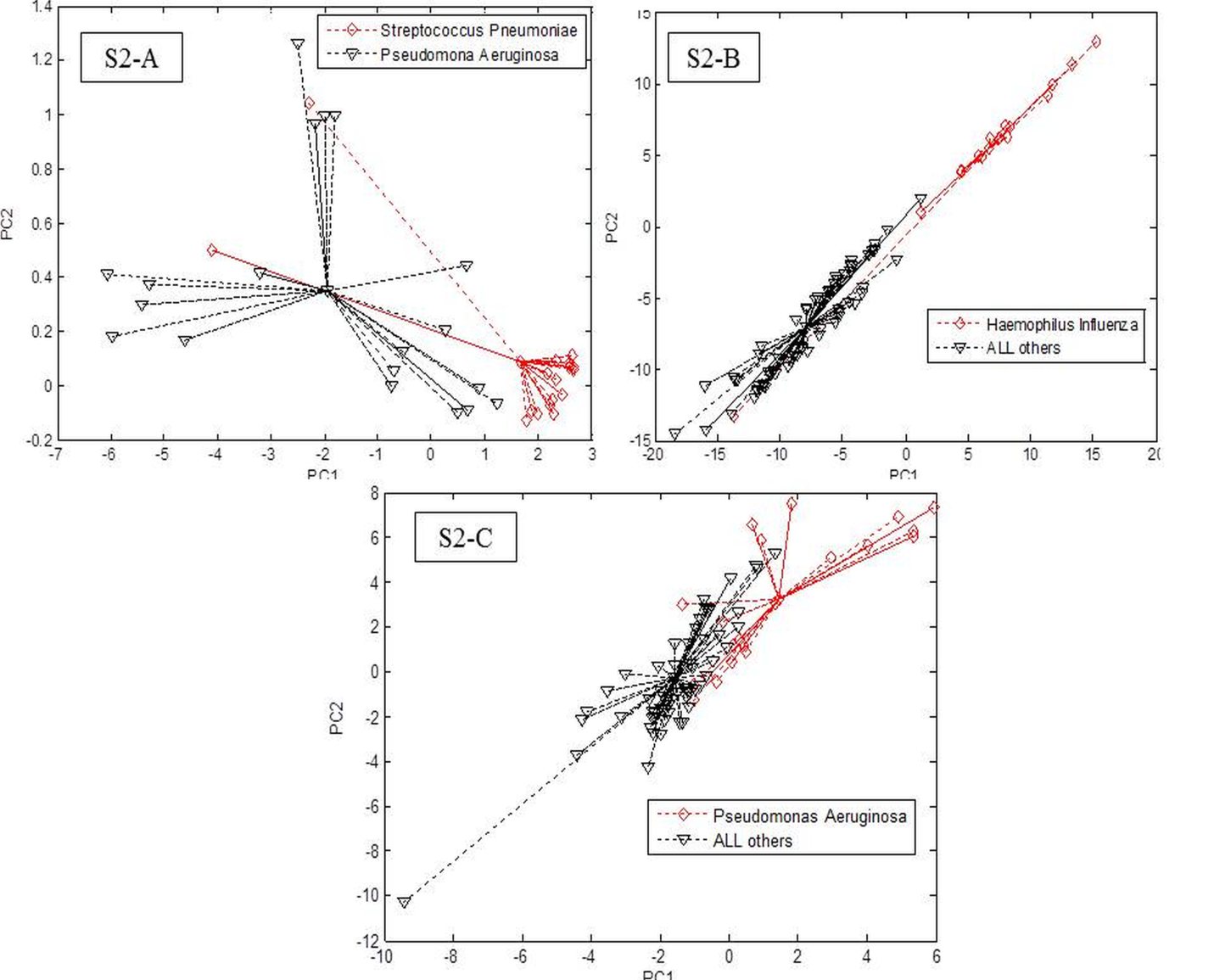


Fig. B
